# Health Commandments

**Published:** 1895-06

**Authors:** 


### SELECTIONS.

### Health Commandments.

*From the Medical and Surgical Age.*

1. Thou shalt have no other food than at meal time.

2. Thou shalt not make unto thee any pies, or put into the pastry the likeness of anything that is in the heavens above or in the earth below. Thou shalt not fail to chew it or digest it, for dyspepsia shall be visited upon the children to the third generation of them that eat pie, and long life and vigor upon those that live prudently and keep the laws of health.

3. Remember thy bread to bake it well, for he will not be kept sound that eateth his bread as dough.

4. Thou shalt not indulge sorrow or borrow anxiety in vain.

5. Six days shalt thou wash and keep thyself clean, and the seventh day thou shalt take a great bath, thou and thy son, thy daughter, and thy maid servant, and the stranger that is within thy gates. For in six days man sweats and gathers filth and bacteria enough for disease; whereupon the Lord has blessed the bath-tub and hallowed it.

6. Remember thy sitting-room and bed-chamber to keep them well ventilated, that thy days may be long in the land.

7. Thou shalt not eat hot biscuit—wait.

8. Thou shalt not eat meat fried.

9 Thou shalt not eat thy food unchewed, or highly spiced, or just before work or just after it.

10. Thou shalt not keep late hours in thy neighbor's house, nor with thy neighbor's wife, nor man servant, nor his maid servant, nor his cards, nor his glass, nor with anything that is thy neighbor's.

Thus endeth the tenth commandment.—*Medical Brief.*

And their are others like unto them, viz:

Thou shalt keep on friendly terms with thy stomach and bowels, for in them abideth more than half the ills of life.

Thou shouldst put nothing in them that would give offence, for they are sensitive members of your ventral family, so to speak, and will not hesitate to call you when you least expect, chiefly in the small hours of the night.

Finally, Thou shouldst measure thy days and nights by these golden precepts, so that in the observance thereof thou mayest keep away from the breakers on the other shore.

### The President of the American Medical Asssociation.

Doctor Donald Maclean has been selected to fill the highest office within the gift of the American Medical Association. This is an honor worthily bestowed, and the Doctor, the Association, and Detroit are alike to be congratulated.—*The Medical Age.*

